# Diagnostic approach to pleural diseases: new tricks for an old trade

**DOI:** 10.12688/f1000research.11646.1

**Published:** 2017-07-17

**Authors:** Fabien Maldonado, Robert J. Lentz, Richard W. Light

**Affiliations:** 1 Division of Allergy, Pulmonary and Critical Care Medicine, Vanderbilt University School of Medicine, 1161 21st Avenue South, T-1218 Medical Center North, Nashville, TN, 37232, USA

**Keywords:** pleural disease, thoracic ultrasound, pleural fluid analysis, clinical prediction model, pleuroscopy

## Abstract

The burden of pleural diseases has substantially increased in the past decade because of a rise in the incidence of pleural space infections and pleural malignancies in a patient population that is older and more immunocompromised and has more comorbidities. This complexity increasingly requires minimally invasive diagnostic options and tailored management. Implications for patients are such that the limitations of current diagnostic methods need to be addressed by multidisciplinary teams of investigators from the fields of imaging, biology, and engineering. Ignored for a long time as an epiphenomenon at the crossroad of many unrelated medical problems, pleural diseases are finally getting the attention they deserve and have spurred a vibrant and exciting field of research.

## Introduction

The term pleura refers to the membrane separating the lungs from the chest wall, which is constituted by two leaflets attached to the outer surface of the lung (visceral pleural) and the inside of the chest wall (parietal pleura), joining at the hilum of the lungs, hence forming a genuine sac surrounding both lungs. The small amount of pleural fluid separating visceral from parietal pleural membranes serves as a lubricant, optimizing respiratory mechanics by facilitating synchronous chest wall and lung movements during respiratory efforts ^1^. Interestingly, surgical removal of the pleura, or chemical fusion of the visceral and parietal membranes for therapeutic purposes (pleurodesis), seems to have little physiologic effect on lung function or work of breathing in long-term human studies and this raises the question of why such a complex structure was selected for in the first place ^2– 5^.

Notwithstanding this lack of clear physiologic function, pleural disorders represent one of the most common reasons for consultation of lung specialists, and an estimated 1.5 million new pleural effusions identified every year in the US alone are caused by more than 60 distinct disease processes ^6^. Approximately 150,000 of these effusions are caused by malignancy—lung and breast cancer are by far the most common offenders—generally due to parietal pleural involvement in advanced disease ^7^. Other common causes of pleural effusions include congestive heart failure, pleural space infections, pulmonary embolism, and manifestations of connective tissue diseases such as lupus or rheumatoid arthritis. It should be intuitively obvious, given the profound implications of such diagnoses for patients, that timely and accurate diagnosis of unexplained pleural effusions is of considerable importance. Pleural fluid aspiration followed by application of the time-honored Light’s criteria remains a remarkably useful step in the diagnostic algorithm of pleural effusions, although an expanding panel of diagnostic tools in the past decade has slowly transformed our approach to pleural diseases, the increasing complexity of which has motivated the development of multidisciplinary pleural subspecialty programs at many institutions in recent years ^8– 12^. In this brief report, we will attempt to summarize what we believe are the most significant and promising recent advances in the diagnosis of pleural diseases and highlight current research efforts in the field.

## 1. Diagnosis of pleural diseases: non-invasive studies

### a. The ultrasound revolution

Thoracic ultrasound, widely available in the form of increasingly sophisticated and affordable portable and handheld units, has unquestionably changed the paradigm in bedside medical evaluation. Basic ultrasound education now complements traditional physical examination training at many medical schools, residency, and fellowship programs. Point-of-care ultrasound for diagnosis and management of thoracic diseases in particular is recommended and endorsed by most relevant scientific societies, and there are established training and certification standards ^13– 16^. In fact, the evidence for improved outcomes and reduced complication rates with ultrasound for thoracic procedures is so overwhelming that its use is now universally considered the standard of care ^17, 18^. Bedside ultrasound examination of the pleura can easily confirm the presence of pleural fluid when conventional chest x-ray findings are equivocal. In addition, it allows an estimation of its volume and complexity and occasionally identifies pleural nodules or masses suggestive of pleural malignancy ^19^. While most physicians use ultrasound merely to mark the site for needle insertion before setting up for the procedure, real-time ultrasound using a sterile sleeve allows sampling of previously unreachable small loculated effusions and occasionally allows parietal pleural biopsies. Furthermore, recent evidence suggests that pre-procedural identification of the intercostal artery, the course of which can be unpredictable, is easily achieved with a high-frequency probe and could help avoid rare but potentially fatal bleeding complications ^20– 22^. Ultrasound examination immediately after the procedure can indirectly suggest the occurrence of pneumothorax by the disappearance of the typical “sliding” sign, representing the sliding of visceral and parietal pleura over each other. Rare but potentially serious bleeding complications from injury of the intercostal artery or one of its collateral vessels may also be identified in the form of rapid re-accumulation of echogenic pleural fluid ^23, 24^.

Interestingly, there has been relatively little research on pleural ultrasound as a predictive tool. Two small studies suggest that a highly organized pleural space with loculations and septations during pleural space infection predicts poorer outcomes, but these studies are small and unadjusted for possible confounders ^25, 26^. An intriguing recent report suggests that ultrasound may allow the identification of unexpandable lung before thoracentesis. In some cases, such as when chronic inflammatory effusions have resulted in the creation of a thick peel around the lung, fluid drainage is not accompanied by lung re-expansion. The vacuum generated can expose patients to complications including pain, pneumothorax, and re-expansion pulmonary edema. Transmission of the heartbeat results in different lung movement and deformation changes that can be identified by using specific ultrasound modes such as the M-mode and speckle tracking ^27^. In this small study, ultrasound in fact performed better than pleural manometry, long regarded as the gold standard for the diagnosis of unexpandable lung and the utility of which is being assessed in an ongoing randomized clinical trial (ClinicalTrials.gov identifier NCT02677883).

### b. Other imaging modalities

There have been comparatively less recent data on other imaging modalities. Whereas loculations (sequestrations of fluid in non-dependent areas) are well identified on computed tomography, the degree of organization within the effusion is better appreciated on ultrasound. In addition, the presence of any of the four following radiologic signs is highly predictive of malignancy: parietal pleural thickening of more than 1 cm, thickened mediastinal pleura, circumferential thickening, and the presence of pleural nodules ^28^. However, in the absence of such specific findings, computed tomography appears neither sensitive nor specific enough to obviate the need for invasive diagnostic interventions ^29, 30^. Positron emission tomography is often used to assess the probability of malignant pleural effusion but with modest sensitivity and specificity, which in a recent meta-analysis were estimated to be 81% and 74%, respectively ^31^. Recently, magnetic resonance imaging has been proposed as a potentially superior imaging modality for pleural effusions in general and malignant pleural mesothelioma in particular but remains largely underutilized compared with computed tomography ^32– 34^. Radiomic approaches to thoracic diseases represent an early and exciting field in imaging science, and very preliminary work in quantitative imaging for the diagnosis of malignant pleural effusion—and staging and diagnosis of mesothelioma in particular—is generating considerable interest ^35, 36^.

## 2. Diagnosis of pleural diseases: minimally invasive studies

### a. Pleural fluid analysis

The “diagnostic separation of transudates and exudates” using a combination of pleural fluid protein and lactate dehydrogenase levels was published in 1972 (in a study that has been cited more than 1,500 times) and to this day remains the single most important step in determining the etiology of a pleural effusion ^11^. Briefly, at the risk of oversimplifying complex processes, transudative effusions typically result from local alterations of the Starling rules that govern capillary microcirculation in the parietal pleura with increased hydrostatic (for example, heart failure) or decreased oncotic (for example, liver failure) pressures, whereas exudative effusions suggest a disease process involving the parietal pleura itself, whether from malignancy, infection, or inflammatory diseases. However, these processes are complex and overlaps are frequent in terms of both pathophysiology and pleural fluid analysis. For example, an estimated 25% of effusions due to congestive heart failure may classify as exudates (“pseudo-exudates”), particularly if the patient is on diuretics ^37^, and conversely a small percentage of documented malignant pleural effusions may classify as transudative effusions. While a high index of suspicion should lead to pleural biopsies in the latter scenario, recent data suggest that the pleural fluid–to–serum albumin gradient could help clarify the etiology of heart failure–related pseudo-exudative pleural effusions ^38^. Pleural and serum brain natriuretic peptide and amino-terminal pro-brain natriuretic peptide levels are also useful to establish heart failure as the cause of the effusion ^39^. Pleural space infections, potentially life-threatening conditions, are usually characterized by typical pleural fluid biochemistry and hence are more straightforward from a diagnostic standpoint, and an interesting recent observation is that the yield of pleural fluid cultures can be increased by 20% by simply inoculating blood culture bottles with pleural fluid at the bedside rather than sending the fluid directly to the laboratory ^40^.

### b. Cytology and biomarkers

The diagnostic utility of pleural fluid analysis for malignant pleural effusions has been the object of several recent studies. The sensitivity of cytology for malignancy is estimated around 60%, and there is a 15% increase with a second procedure ^41^, although some reports suggest much lower estimates ^42^. Others focusing on modern immunohistochemistry techniques seem much more optimistic, particularly for mesothelioma, a primary pleural malignancy which traditionally requires pleural biopsies for definitive diagnosis ^43, 44^. In addition, pleural fluid has been shown to be a biospecimen suitable for the majority of molecular analyses required for targeted therapy ^45^. High-throughput techniques and modern molecular techniques promise to identify biomarkers expressed by cancer cells or their environment that could ultimately transform our diagnostic approach. Novel proposed techniques include single-cell mechanophenotyping which evaluates the deformability of pleural cells ^46^, circulating tumor cells and cell-free tumor DNA ^47, 48^, and metabolic-based assays to identify non-leukocyte metabolically active tumor cells ^49^. These recent developments, though exciting, remain preliminary and are still closer to the bench than they are to the clinic. The search for optimal biomarkers has been hampered by a lack of standardized methodology and failure to externally validate promising results, as in the case of fibulin-3, a biomarker once anticipated to transform our approach to malignant mesothelioma ^50– 53^. Large ongoing research projects are attempting to identify reliable alternative candidates.

### c. Clinical prediction models

An unprecedented number of well-designed randomized controlled studies published in the last decade by a growing international pleural research network have clarified and sometimes transformed patient management for malignant pleural effusions and pleural space infections in particular. However, one major obstacle faced by researchers and clinicians has been the lack of clinical prediction models allowing appropriate patient selection and stratification. The RAPID (renal, age, purulence, infection source, and dietary factors) score was derived and validated from two large datasets and proposes to risk-stratify patients with pleural space infections, and ultimately its purpose is to individualize management, which currently is subject to local preferences and expertise rather than patient characteristics ^54^. A large multicenter observational prospective study to validate this score is ongoing. Similarly, the LENT (pleural fluid lactate dehydrogenase, Eastern Cooperative Oncology Group performance score, neutrophil-to-lymphocyte ratio, and tumor type) score is a clinical prediction model that provides estimates of survival for patients with malignant pleural effusions, which could prove very useful in subject selection in clinical trials and ultimately individualized medical or surgical management of pleural effusions ^55^.

## 3. Diagnosis of pleural diseases: invasive studies

Approximately 25% of exudative pleural effusions remain without identifiable causes after pleural fluid analysis and cytology. Parietal pleural biopsies are the recommended next step in the diagnostic assessment but often are not pursued as they sometimes require thoracic surgery which, though less invasive since the advent of video-assisted thoracoscopic surgery, remains a major operation requiring general anesthesia, double-lumen endotracheal intubation, large-bore chest tube placement, and a hospital stay. Hence, clinicians often default to observation after an unfruitful pleural fluid analysis ^56^. An example from a Bayesian approach to diagnosis might be helpful to illustrate the limitations of this approach. Given a sensitivity for cytology of 60% and a specificity of 100%, a patient with a 50% pre-test probability of pleural malignancy would have, after a negative cytology, a remaining 27% post-test probability of disease. After a second thoracentesis, this number decreases to only approximately 18% (a generous estimate as the same test is used twice). Given the profound implications of a diagnosis of malignant pleural effusion, it should be intuitively obvious that moving forward with additional, ideally minimally invasive, diagnostic tests would be desirable.

Reclaiming a procedure initially introduced by Hans Christian Jacobeus, a Swedish Professor of Internal Medicine at the Karolinska Institute in Stockholm from 1916 to 1937, interventional pulmonologists and pleural specialists have popularized minimally invasive, so-called “local anesthetic” pleuroscopy, which allows pleural exploration and biopsies in awake patients and often is performed on an outpatient basis ^57– 61^. While pleuroscopy is slowly gaining traction in the US, centers offering the procedure have exponentially increased over the past decade in Europe and other parts of the world and presumably this is due to more favorable regulatory environments and sometimes more selective access to thoracic surgery. The development of dedicated interventional pulmonology training programs in the US, with accreditation standards that include pleuroscopy training endorsed by all relevant medical societies, may facilitate a more widespread adoption of this safe and effective diagnostic modality ^15^. Even less invasive interventions, such as “mini-thoracoscopy”, using increasingly smaller instruments, have been proposed ^62^.

When focal pleural lesions can be identified by computed tomography or ultrasound, percutaneous image-guided biopsies performed by interventional radiologists or pulmonary specialists are a useful and minimally invasive approach to diagnosis which in recent studies has had a yield similar to that of thoracoscopy but without the option to offer definitive treatment in the same setting (pleurodesis or indwelling pleural catheter placement) ^63^.

## Conclusions

The rising burden of pleural disease in an increasingly complex patient population demands a more tailored approach to diagnosis and management than ever before. Thoracic ultrasound, the application of new bioassays in addition to foundational biochemical analysis of pleural fluid, the development of models for prognosis and prediction of treatment response, and the resurgence of medical thoracoscopy-pleuroscopy comprise recent advances in pleural disease, and there is a great need for further basic, translational, and clinical research in this field.
